# Transcutaneous Vagus Nerve Stimulation (tVNS) for headache prophylaxis: initial experience

**DOI:** 10.1186/1129-2377-14-S1-P198

**Published:** 2013-02-21

**Authors:** D Magis, P Gérard, J Schoenen

**Affiliations:** 1 University Department of Neurology, Belgium

## Introduction

Neurostimulation is of increasing interest in headache therapy. Invasive neurostimulation methods were found effective in drug-refractory headaches. There is a need for non invasive therapies that could be justified in less disabled patients. Previous case reports suggested that internal Vagus Nerve Stimulation (VNS) might be effective in headache prevention [1-3].

## Objectives

We explored efficacy and safety of a transcutaneous VNS device (tVNS, Gammacore®) as preventive treatment in primary headache sufferers. The aim of this pilot trial was to determine a target subpopulation for a multicenter sham-controlled study.

## Methods

Eighteen patients accepted to undertake prophylaxis with tVNS: 12 migraine without aura patients (MO, 5 with medication overuse, MOH, 3 chronic), 4 patients with trigeminal autonomic cephalalgia (2 chronic cluster headache, CCH), and 2 with hemicrania continua (HC). tVNS was applied 3 times/day during 90 seconds. Data were collected using headache diaries.

## Results

Results are available for 13 patients. Ten patients stopped tVNS after 0.7 to 6 weeks because of lack of efficacy (N=9) and/or side effects (N=6). In one patient with CCH, attacks decreased from 4.5/day to 0.39/day, and in a patient with MOH headache days decreased from 7/week to 3/week and intensity from 8.5 to 4/10. The benefit remains after 5 months of treatment and attack frequency increases when stimulation is interrupted. The last patient had HC with initial intensity decrease from 8.5 to 4/10 but relapsed after 8 weeks. Reported side effects were local discomfort (N=3), tonic muscle contraction (N=3), fatigue (N=1), palpitations (N=1) and cervical muscle spasm (N=1).

## Conclusions

Our initial experience suggests that tVNS might help some headache patients. That it was not effective in many patients may be due to the fact that the vagus nerve is not or insufficiently stimulated. There were no serious adverse events but the stimulation could be poorly tolerated.
